# Isolated APTT prolongation—not always a bleeding risk in acute paediatric burns surgery

**DOI:** 10.1007/s00238-014-1012-y

**Published:** 2014-09-05

**Authors:** M. Nizamoglu, K. S. Alexander, U. Anwar, S. Bhandari

**Affiliations:** Pinderfields General Hospital, Mid Yorkshire NHS Trust, Wakefield, WF1 4DG UK

Sir,

There are several causes of isolated elevated activated partial thromboplastin time (APTT), some of which are known to cause increased bleeding. This case report demonstrates not all causes of elevated APTT cause increased bleeding during acute burns surgery.

A 2-year-old Indian girl was admitted to the burns unit the same day following a slip into freshly boiled water from which she sustained 6 % TBSA mixed partial/full-thickness scald of her left thigh and buttock. No immediate first aid was administered. There was no relevant past medical or family history.

During her stay, she developed tonsillitis. Blood results showed the following: haemoglobin 12.8 g/L (normal 11.0–13.8), white blood count 7.4 × 10^9^/L (6–17) and C-reactive protein 229 mg/L (0–9). Intravenous benzylpenicillin and flucloxacillin and benzydamide hydrochloride 0.15 % mouthwash were administered. Later, pruritic petechial spots developed on her face and limbs. Following paediatric review, coagulation screen and varicella zoster virus serology were performed. APTT was raised at 87 s. Prothrombin time (PT) and fibrinogen levels were normal. After the haematologist advised blood cultures, meningococcal/pneumococcal PCR; viral screens and clotting factor 8, 9, 11 and 12 assays were tested and shown to be normal. Her APTT was 185 s when repeated using the SynthASil reagent, which has greater sensitivity to contact factor deficiencies. APTT synthetic phospholipid (SP), a reagent sensitive to lupus anticoagulant, was mildly elevated at 55.8 (normal value 25–38). Dilute Russell’s viper venom time (DRVVT), a test used for the detection of lupus anticoagulant and antiphospholipid antibody including anticardiolipin, was normal. Despite no clinical wound infection, wound swabs grew MRSA, which was treated with mupirocin 2 % ointment. The consultant paediatric haematologist advised her results suggested a contact factor deficiency, which would not impair haemostasis as there was no coagulopathy clinically. The burn was debrided under general anaesthetic using Versajet hydrosurgery. Haemostasis was aided using topical adrenaline swabs. The donor site was infiltrated using 1:500,000 adrenaline/saline solution. A split-thickness skin graft 6/1,000 of an inch was harvested from her posterior left thigh using a dermatome. The graft was meshed to 1.5:1 ratio then applied to the wound using tissue acrylic. No further blood-sparing techniques were required. There were no intra/postoperative complications or evidence of blood loss greater than expected. Blood loss of 50–100 ml was observed. Blood transfusion was not required.

There are several conditions resulting in an isolated prolonged APTT. Deficiencies in contact factors, factors 8, 9, 11 and 12 deficiencies, all result in an isolated APTT prolongation, as well as lupus anticoagulant, anticardiolipin, Von Willebrand disease and acquired clotting factor inhibitors such as acquired haemophilia A. Shah et al. in 2006 [1] looked at 90 subjects between the ages of <1 and 18 referred with a prolonged APTT. Among the subjects, 48 % had no clear aetiology; 23 % due to lupus coagulant; 13 % due to factors 8, 9, 11 and 12 deficiencies; 12 % due to anticardiolipin; 11 % Von Willebrand disease and 3 % others. Of these causes, only factor 8 (haemophilia A), factor 9 (haemophilia B) and factor 11 (haemophilia C) deficiencies and Von Willebrand disease cause clinically significant bleeding disorders. Shah et al. concluded a raised APTT had a positive predictive value of bleeding complications only in the presence of clinical symptoms or a documented family history.

While contact factors prekallikrein (PK) and high molecular weight kininogen (HMWK) are required for normal APTT, they are not essential for normal coagulation to occur. Patients with these deficiencies have no apparent bleeding tendency. This may be because proteins of the contact system play a secondary role in thrombin generation. Such factor deficiencies do not result in a marked bleeding, as the majority of factor 11 is activated via platelets and thrombin [2, 3]. It is probable that most of the patients with PK deficiency are asymptomatic and go unrecognized [4]. Most patients are diagnosed incidentally [3]. From the current literature available, there does not appear to be any proven link between contact factor deficiencies and an increased bleeding or prothrombotic risk [5, 6]. Several cases of isolated HMWK deficiency have been reported in the literature undergoing surgery without bleeding complications [7, 8].

In the case of an elevated APTT in an acute burns patient, it is important to determine the underlying cause and whether this causes increased bleeding. Suspected cases can be confirmed by assessing serum contact factor levels. This was not available in our case as our test sample haemolysed; however, this is recommended to confirm the diagnosis. If contact factor deficiency is responsible for the prolonged APTT, there is no increased perioperative bleeding risk unless there is a past medical/family history of excessive bleeding. This must be considered prior to any operative intervention. Specialist haematological opinion is advised.

This case illustrates that an isolated prolonged APTT does not necessarily cause a bleeding tendency during burn debridement and skin grafting surgery. Knowledge of these causes is of importance to the burns team and should be taken into consideration along with clinical symptoms, any previous history of a bleeding tendency and family history before proceeding with surgery.
